# A Retrospective Single-Arm Cohort Study in a Single Center of Radiofrequency Ablation in Treatment of Chronic Radiation Proctitis

**DOI:** 10.3390/life13020566

**Published:** 2023-02-17

**Authors:** Chien-En Tang, Kung-Chuan Cheng, Kuen-Lin Wu, Hong-Hwa Chen, Ko-Chao Lee

**Affiliations:** Division of Colorectal Surgery, Department of Surgery, College of Medicine, Kaohsiung Chang Gung Memorial Hospital, Chang Gung University, Kaohsiung 83301, Taiwan

**Keywords:** chronic radiation proctitis, radiofrequency ablation, pelvic radiotherapy

## Abstract

Background: Chronic radiation proctitis (CRP) may develop in patients within months to years after undergoing pelvic radiotherapy. Numerous treatment modalities are available to achieve hemostasis in CRP, but the optimal treatment remains controversial. We report our clinical experience and long-term outcomes using radiofrequency ablation (RFA) in patients with CRP. Methods: We retrospectively reviewed patients who underwent RFA for CRP at Kaohsiung Chang Gung Memorial Hospital between October 2015 and March 2021. The patient characteristics, endoscopic findings, and clinical outcomes were collected and analyzed. Results: 35 total patients were enrolled in the study. The mean age was 70.5 ± 12.4 years. All patients sustained repeated rectal bleeding before RFA, and 15 of 35 patients needed blood transfusion. Bleeding cessation was achieved in all patients. Mean follow-up time was 18.6 months (ranging from 2 to 52 months). The hemoglobin (Hb) levels at 6 months after RFA revealed significant improvement from 11.0 ± 2.3 to 11.8 ± 1.9 g/dL (*p* = 0.048). The rectal telangiectasia density (RTD) scores also showed significant improvement from 2.96 ± 0.2 to 0.85 ± 0.7 (*p* < 0.0001). In conclusion, RFA treatment is safe and effective in controlling rectal bleeding in CRP without serious complications and can be considered as a first-line or alternative endoscopic treatment for patients with CRP.

## 1. Introduction

In the last few decades, pelvic radiotherapy has been used as a common treatment modality for pelvic malignancies. Despite the survival benefits and improved clinical outcomes associated with pelvic radiotherapy, it carries numerous side effects that would result in a decline in the patient’s quality of life [1,2,3]. Radiation proctitis (RP) is one of the most common complications of pelvic radiotherapy. RP can be acute or chronic based on the time from initiation of radiotherapy to the development of presenting symptoms. Acute RP is an inflammatory reaction to radiation and mainly occurs immediately or within the first three months after pelvic radiotherapy. The symptoms of acute RP include diarrhea, anorectal pain, or rectal bleeding, and these are usually self-limiting and most resolve within two to six months. Chronic radiation proctitis (CRP) occurs in 5–20% of patients with more severe symptoms and is often associated with numerous complications [4,5,6]. CRP results from damage to rectal epithelium, microvascular injury with intimal fibrosis, and chronic ischemia caused by radiotherapy. In contrast to acute complications, CRP is considered a progressive condition and a significant source of morbidities. The symptoms of CRP are similar to acute complications, including diarrhea, mucoid discharge, tenesmus, rectal pain, and rectal bleeding. These symptoms may resolve spontaneously or remain asymptomatic in many cases. However, some patients may suffer from persistent rectal bleeding that would require repeated blood transfusion [2,3,7,8].

According to disease severity, numerous treatment options are available for CRP, including medical, endoscopic, and surgical treatments. Generally, medical treatments are considered as first-line management, including formalin irrigation, topical steroid application, hydrocortisone enema, or hyperbaric oxygen (HBO) therapy. Endoscopic treatments, including argon plasma coagulation (APC) and radiofrequency ablation (RFA), are reserved for more severe or refractory patients [2,3,9,10]. However, there is no consensus on the optimal treatment for repeated rectal bleeding in CRP. Currently, APC is widely used and considered as the first-line endoscopic therapy for CRP. APC provides non-contact electrocauterization with high-frequency energy conducted by ionized gas. The coagulation depth (ranging from 0.5 mm to 3 mm) is controllable and achieves adequate hemostasis. Despite the benefits of APC, it has limited effects for severe CRP with extensive rectal surface bleeding, and the treatment may cause some major complications including perforation and extensive necrosis in 14% of patients [11].

Endoscopic RFA is an alternative endoscopic treatment for CRP and is effective and safe for controlling rectal bleeding in CRP [9]. RFA is characterized by focused-energy penetration and restricts damage to superficial rectal mucosa with broad fields of ablation. It also leads to fewer major complications, such as perforation or stricture, that are associated with other interventions [12]. The aim of this study is to describe the clinical outcomes and long-term follow-up results of a large case series, with patients in a single medical center in Taiwan who underwent RFA for refractory rectal bleeding of CRP.

## 2. Methods

### 2.1. Ethical Considerations

After obtaining approval (approval number: 202300054B0) from the institutional review board (IRB) of Chang Gung Memorial Hospital, we reviewed patients who underwent RFA for CRP at Kaohsiung Chang Gung Memorial Hospital between October 2015 and March 2021.

### 2.2. Patient Population

A total of 35 patients with medical history of pelvic radiotherapy and outpatient referrals for repeated rectal bleeding were included in this study. Inclusion criteria were patients who had previously undergone pelvic radiotherapy for malignant diseases including prostate, cervical, and rectal cancers and were suffering from recurrent hematochezia with or without medical treatment. All patients underwent digital rectal examination and colonoscopic evaluation before treatment, and patients with rectal tumors, recurrent malignancies, radiation sigmoiditis, anorectal stricture, anal incontinence causing treatment intolerance, or coagulopathy were excluded. Patient characteristics were collected for analysis, including age, sex, Eastern Cooperative Oncology Group (ECOG) performance status, comorbidities, details on pelvic malignancy and treatment, previous treatments for CRP, need for blood transfusion, post-RFA laboratory results, and endoscopic findings. We used the rectal telangiectasia density (RTD) grading scale to evaluate CRP severity. The RTD scoring scale is as follows: Grade 0—normal mucosa; Grade 1—less than 10 discrete telangiectasias; Grade 2—a single coalescing patch and/or >10 discrete telangiectasias; Grade 3—more than 2 coalescing patches. High RTD score is associated with hematochezia [13]. The pre-treatment RTD score was obtained before RFA treatment. The post-treatment RTD score was obtained at the last follow-up with colonoscopic examination.

### 2.3. Radiofrequency Ablation Protocol

**Figure 1:**
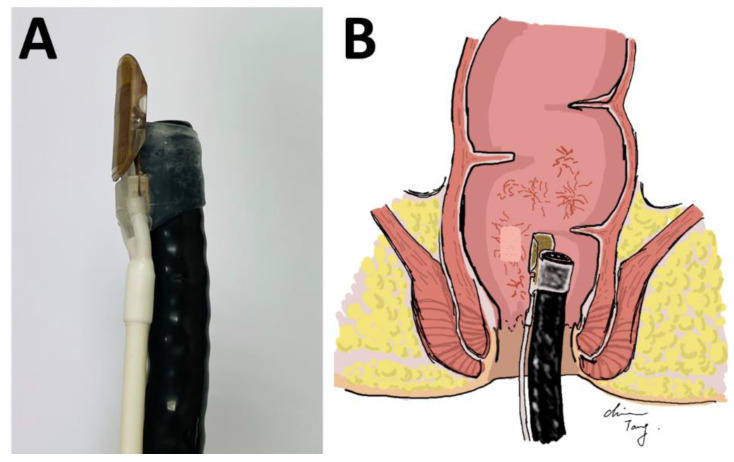
The Halo^90^ radiofrequency ablation system. The Halo^90^ RFA electrode paddle and catheter were mounted on a colonoscope at the 12 o’clock position. (**A**). A 13-mm-wide by 20-mm-long bipolar electrode array was on the dorsal surface of the platform. (**B**). A 15 J/cm^2^ RFA was applied twice at each telangiectasia with active bleeding site to achieve adequate ablation and hemostasis. RFA, radiofrequency ablation.

Before RFA treatment, all patients underwent standard bowel preparation (using sodium picosulfate or polyethylene glycol) to minimize stool contamination and to obtain an adequate operating field. The RFA platform was mounted on the colonoscope at the 12 o’clock position with an electrode array positioned on the colonoscope cap (HALO^90^ system; Covidien GI Solutions, Sunnyvale, CA, USA). The RFA platform was 30-mm radius in size, with a bipolar electrode array fixed on the dorsal surface. After inserting the RFA device into the patient’s rectum, the operator inspected and identified the bleeding areas caused by telangiectasia and applied the RFA electrode paddle to the target area. According to current suggestions [14], in order to achieve effective eradication of focal areas of CRP, each lesion site should be treated twice with the ablation energy setting of 12 or 15 J/cm^2^ and power setting of 40 W/cm^2^. Generally, we performed 1–2 RFA applications for each telangiectasia site to achieve adequate ablation and hemostasis (Figure 1). We used an energy setting of 15 J/cm^2^ for telangiectasia sites with active bleeding within 8 cm above the dentate line, and an energy setting of 12 J/cm^2^ for those in the middle to upper rectum, which is over 8 cm above the dentate line. No sigmoid colon lesion was treated in consideration for safety concerns, in particular to avoid perforation, since the wall of the sigmoid colon is thinner than that for the rectum. The energy was delivered and terminated automatically by the Halo^90^ system after we applied the RFA electrode paddle to the rectal mucosa. Resultant coagulum was not removed from the treatment area. For telangiectasia near the dentate line, we used the retroflexion technique to perform RFA. The colonoscope tip and RFA paddle were deflected in a U-turn, allowing clear visualization and application. We avoided circumferential ablation in concern of stricture formation. After ablation, we switched to another colonoscope without a RFA probe for irrigation and inspection. We then reinserted the colonoscope with the RFA probe to treat another telangiectasia site and to achieve adequate hemostasis. If complete coagulation was not achieved, we applied another 1–2 additional treatments with an energy setting of 12 J/cm^2^.

After RFA treatment, we recorded the patient’s condition, including hemoglobin (Hb) level, RFA-induced complications, number of emergency room (ER) visits related to rectal bleeding, need for blood transfusions, and endoscopic severity score. We arranged colonoscopic evaluation and checked Hb levels at the 1st, 3rd, 6th, and 12nd months after RFA treatment. Retreatment was considered in patients with continuous rectal bleeding and severe anemia after 12 to 16 months since initial RFA therapy.

## 3. Statistics

We used the IBM SPSS software from Windows, version 26.0 (IBM Corp., Armonk, NY, USA) for statistical analysis. Continuous data are expressed as mean ± standard deviation and analyzed using the paired Student’s *t*-test. A *p*-value of <0.05 was considered to be statistically significant.

## 4. Results

### 4.1. Population Characteristics

A total of 35 patients, including 27 (77.1%) males and 8 (22.9%) females that underwent RFA for CRP at Kaohsiung Change Gung Memorial Hospital between October 2015 and March 2021, were enrolled in this retrospective study. Patient characteristics are shown in Table 1 and Appendix A. Patient mean age was 70.5 ± 12.4 years. The majority of patients had an ECOG performance status of 0 or 1 (94.3%). All patients received pelvic radiotherapy for pelvic malignancies, and the primary cancer sites were 25 (71.4%) prostate cancers, 8 (22.9%) cervix cancers, and 2 (5.7%) rectum cancers. The prescription dose was 5000–7500 cGy in 32 (91.4%) patients. Three (8.6%) patients received radiation doses less than 5000 cGy, and two patients (5.7%) received short-course proton beam radiotherapy. The onset interval of CRP after radiotherapy completion was 12.3 ± 6.5 months. Thirty-one (88.6%) patients had previously received either medical or endoscopic therapy prior to RFA. All patients had repeated rectal bleeding, and 15 (42.9%) required blood transfusion. Repeated rectal bleeding not only led to decline in quality of life of patients, but also resulted in life-threatening events, in some cases.

All RFA procedures were performed by a single surgeon to minimize technical bias. All patients regularly visited the outpatient department for follow-up after RFA procedure. Thirty-four (97.1%) patients received only one session of RFA. One patient had an incomplete RFA procedure the first time due to patient intolerance and received a second session of RFA procedure 4 months later. Complete cessation of rectal bleeding was achieved in all 35 patients.

The mean follow-up time was 18.6 months, ranging from 2 months to 52 months. Post-RFA Hb level was available for 32 (91.4%) and 28 (80%) patients at 3 and 6 months after treatment. The Hb level improved at 3 months after RFA treatment, but it was not statistically significant. The Hb level significantly improved from 11 ± 2.3 before RFA treatment to 11.8 ± 1.9 at 6 months after RFA treatment (*p* = 0.048). Thirty-four (97.1%) patients had an RTD score of 3 at baseline.

### 4.2. Endoscopic Findings after RFA

**Figure 2:**
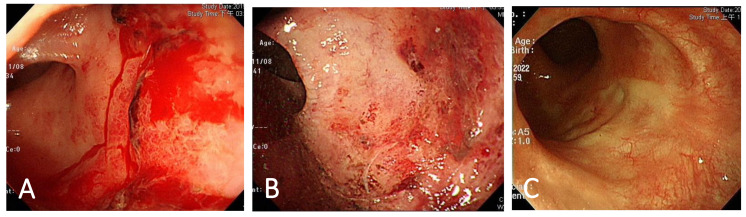
Endoscopic findings of telangiectasia before and after RFA treatment. (**A**) Multi-focal telangiectasia with active bleeding before RFA treatment. (**B**) Adequate hemostasis was achieved immediately after RFA treatment. (**C**) Endoscopic findings showed complete mucosal healing at 3 years after RFA treatment.

Colonoscopic evaluation after RFA treatment was available in 25 (71.4%) patients. There was a significant improvement in mean RTD score from 2.96 ± 0.2 before RFA treatment to 0.85 ± 0.7 (*p* < 0.0001) after RFA treatment (Table 2). The colonoscopic findings from before and after RFA treatment are shown in Figure 2A,B. The multi-focal bleeding telangiectasia site revealed whitening after adequate hemostasis was achieved, immediately after RFA treatment. Figure 2C shows a colonoscopic photograph with complete re-epithelialization at 3 years after RFA treatment. There was no further bleeding and no evidence of new growing telangiectasia.

### 4.3. Adverse Events after RFA

The adverse events that occurred after RFA treatment are shown in Table 3. Of 35 patients, 25 experienced intermittent hematochezia after RFA procedure, and the mean duration of hematochezia was 7.4 ± 4.4 weeks, ranging from 2 to 24 weeks. The most common adverse event was mild to moderate anal pain in 12 (34.2%) patients, which was controlled with oral analgesics (acetaminophen alone or combination with other non-steroidal anti-inflammatory agents) or topical (lidocaine/betamethasone ointment) therapy. Most rectal discomforts subsided after 4 weeks. Two patients had mild anal stenosis, which was managed by finger dilation at the outpatient department. There were seven patients lost to follow-up, including two patients with recurrent primary malignancies. Within 2 months after RFA procedure, two patients visited the ER due to significant hematochezia. Both patients were hemodynamically stable, but one of the patients required blood transfusion to treat anemia. The bleeding was considered to be caused by delayed mucosal re-epithelialization after RFA treatment and would stop spontaneously after complete mucosa re-epithelialization. Though one significant rectal bleeding event was noted within 2 months after RFA, all patients experienced significant improvement in clinical symptoms without further need for blood transfusion at their last outpatient department visit.

## 5. Discussion

In this series of 35 patients, RFA treatment was effective and well tolerated in all patients and did not lead to any major complications at the last outpatient department visit. However, one hemodynamically stable, but significant, post-RFA rectal bleeding was still noted within 2 months, which required a blood transfusion. Our results showed significant improvement in clinical symptoms and Hb level and a reduction in the need for blood transfusion. To our knowledge, this is the largest case series on RFA treatment for refractory rectal bleeding of CRP in Taiwan and Asia. In addition, this study includes not only patients with prostate cancer, but also cervical cancer and rectal cancer patients.

Pelvic radiotherapy leads to rectal mucosal injuries and dysfunction at the baseline of the mucosal microvasculature, which causes chronic ischemia and fibrotic changes of the rectum [15,16]. In addition, the increased expression of the vascular endothelial growth factor in response to radiation damage could lead to increased vascular telangiectasia density and result in recurrent rectal bleeding [14]. The incidence of CRP is reported at 2–20%. Unlike acute radiation proctitis, the symptoms of CRP, including severe bleeding, stricture, and obstruction may not become apparent until months to years after radiotherapy, and the mean time of symptom onset is about 8 to 12 months after completion of radiotherapy [6]. Radiation dosages greater than 4500 cGy, hypertension, diabetes mellitus, and a history of pelvic inflammatory disease are thought to be risk factors of CRP [17]. Blacksburg et al. reported a low incidence of grade 3 proctitis in patients who received radiotherapy with 3500 cGy, delivered in five fractions [18]. However, this study reports three patients who received a radiation dosage of less than 5000 cGy and suffered from CRP with repeated rectal bleeding.

There are several noninvasive and invasive treatments available for CRP. Topical use of anti-inflammatory agents such as sulfasalazine, mesalamine, or steroids is usually used as the first-line noninvasive treatment. Anti-inflammatory agents are thought to inhibit prostaglandin synthesis and free radical scavenging activity [6]. However, responses to topical treatments are limited, and small sample size trials have shown disappointing results for topical treatments when used in patients with CRP with refractory rectal bleeding [19]. Thirty-five (85.7%) patients in our study previously received topical treatment and also demonstrated poor response rate, which was compatible with previous studies. Hyperbaric oxygen therapy (HBO) is another noninvasive treatment modality for CRP. HBO therapy inhibits bacterial growth, promotes tissue oxygenation and neovascularization, and reverses the fibroatrophic process induced by radiotherapy [10,20]. A randomized, controlled, double-blind trial demonstrated significant improvement in CRP using HBO therapy, which enhanced patient quality of life and prevented further advanced intervention [21]. However, HBO therapy may not be readily available and lacks a standard protocol in treatment for CRP. In addition, it is an expensive therapy requiring specialized equipment and several weeks of treatment sessions. Topical application of formalin, acting via chemical cauterization, has been reported to be effective in CRP, but it is associated with higher rate of major complications such as stricture or fistula [22].

Endoscopic management is suggested in patients with noninvasive treatment failure or refractory rectal bleeding. APC is widely used for hemorrhagic digestive malformations and is considered the first-line endoscopic treatment for CRP [11]. In previous studies, APC showed high clinical success rates and achieved bleeding cessation in up to 87% of patients, with low complication rates [11,23,24,25]. APC is a non-contact electrocoagulation technique with controllable depth of around 0.5 mm to 3 mm. However, compared to other endoscopic management, APC is disadvantageous due to the risk of colon overdistension caused by the argon gas flow [24]. The complication rate reported after APC treatment is variable. The most common complication is anal or rectal pain, which occurs in 20% of patients and typically subsides spontaneously. Serious complications such as colonic fistula, perforation, explosion, or stricture are reported in 4% of patients [25].

In contrast to APC, RFA is a contact treatment for CRP and provides limited energy penetration, restricting damage to the superficial mucosa and muscularis propria without injuring the submucosa [14]. RFA administered with the HALO^90^ system allows for quick energy delivery (approximately 250 ms) with high-power density (40 W/cm^2^), and results in uniform tissue penetration depth (approximately 1000 μm). The ablation of mucosa using the HALO system provides controlled destruction, whereas the submucosa typically remains uninjured, thereby minimizing possible complications [26]. In a pilot trial, sites treated with RFA at an energy setting of 15 J/cm^2^ demonstrated controlled tissue alteration limited to the muscularis propria and superficial submucosa layers. A higher energy (20 J/cm^2^) setting or a lower energy setting (12 J/cm^2^) with greater than two applications resulted in deeper tissue alteration to the serosa [27]. RFA was originally designed to ablate Barrett’s esophagus and has demonstrated effectiveness and safety in the past few decades [26,28]. In 2009, Zhou et al. reported the first successful experience in treating CRP with RFA. In their study, endoscopic optical coherence tomography (EOCT) arranged in all three cases prior to and after RFA treatment revealed excellent re-epithelialization [14]. In Table 4, we summarize the published studies, including case series, prospective trials, retrospective reviews, and meta-analyses, that evaluate the clinical impact of RFA for CRP [12].

Dray et al. published a study with 17 patients treated with a median of two RFA sessions (range 1–4) with a follow-up period of 6 months [31], and they demonstrated an overall technical success rate of 100% (17/17) immediately after the end of the treatment. In their study, two patients received additional APC treatment after the last RFA session due to persistent rectal bleeding. Rustagi et al. (2015) [12] published the largest study at the time, including 39 patients who underwent RFA for CRP. The authors described an overall technical success rate of 100% (39/39); however, one patient showed no change in endoscopic RTD score. The mean follow-up time was 28 (range: 7–53) months, and complete cessation of rectal bleeding was achieved in all patients. They also showed a satisfactory response rate for those dependent on blood transfusions as 92% of these patients no longer needed blood transfusion for rectal bleeding. In 2018, Chou et al. reported the first case series using RFA treatment in Asian patients [33]. All three patients achieved a satisfactory response, consistent with results from previous studies in western populations. McCarty et al. (2019) [9] enrolled six publications in a systematic review and meta-analysis and evaluated the efficacy and safety of RFA for CRP. Of the 71 patients included in the six studies, overall technical and clinical success rates were 100% and 99%, respectively. For patients who failed to respond to prior endoscopic treatment, RFA still demonstrated a satisfactory response rate and improvement in Hb level. Generally, most studies used an ablation energy setting of 12 J/cm^2^ for RFA applications. One study (*n* = 7) and our study used the ablation setting of 15 J/cm^2^, which also demonstrated a high technical and clinical success rate, without serious adverse events.

A common adverse event of RFA is intermittent hematochezia, which is considered to be caused by the process of mucosal healing at the RFA site. The duration of hematochezia was noted to be 12 to 16 weeks in a previous study [12]. In our study, 25 patients sustained intermittent hematochezia which lasted for 2 to 24 weeks, with a mean of 7.4 weeks. As described in previous studies, squamous re-epithelialization after RFA may be the primary reason why rebleeding is prevented (Figure 2C) [9,12,14,31]. In addition, Ahsen et al. demonstrated that both mucosal and submucosal vasculature were observed to be normalized under optical coherence tomography angiography after RFA [34]. The duration of hematochezia after RFA treatment may be related to the time needed to achieve complete mucosal healing and squamous re-epithelization. Retreatment is suggested 12 to 16 weeks later after initial therapy if patients experience continued rectal bleeding [12]. Other adverse events of RFA include mild to moderate anorectal pain and tenesmus, but most symptoms can be managed with oral analgesic agents and subside spontaneously within a few weeks. No serious complications have been reported in previous studies [12,31,33,35]. Our study demonstrated statistically significant change in mean Hb level; however, the change is not as dramatic as those mentioned in earlier studies [9,12,14,31]. A possible explanation for this is that the majority of patients (88.6%) in our study received previous treatment, which may have resulted in improved baseline Hb levels compared to previous studies. Nonetheless, the high rate of bleeding cessation and discontinued necessity for blood transfusions indicate that RFA is an effective treatment for CRP.

Surgery is considered as a last resort and is reserved for patients in which medical and endoscopic treatments have failed. Intractable rectal bleeding, perforation, obstruction, and fistulas are the main reasons for patients who require surgical intervention. Surgical methods for CRP may vary from fecal diversion to proctectomy with or without anastomosis. However, most studies demonstrated poor outcomes with high complication rates in cases where surgical intervention were indicated [36]. None of the patients in our study required surgical intervention during the follow-up period.

This study had several limitations. First, it was a retrospective single-arm cohort study in a single center and the conclusions were limited by the lack of a control group. Further comparative studies or randomized trials are warranted. Second, most of our patients had received previous intervention before RFA, which may have led to selection bias. Third, the RTD score for endoscopic severity was evaluated by a single surgeon, which may have introduced potential subjective bias. In addition, the lack of objective data on patient satisfaction and symptomatic improvement may have also resulted in potential subjective bias. However, our study showed similar efficacy and safety to the reported literature in western populations.

## 6. Conclusions

In conclusion, our study demonstrated that RFA treatment for CRP is well tolerated and has high clinical success rate and good long-term outcomes without serious complications, even in elderly patients with multiple comorbidities. It should be considered as a first-line or alternative endoscopic treatment of choice for patients with refractory rectal bleeding. Further prospective controlled studies are required to compare RFA with other endoscopic treatments.

## Figures and Tables

**Table 1:** Patient characteristics.

Variable	Results
Total patients, no.	35
Age, mean (±SD), years	70.5 (±12.4)
ECOG performance status	
0	21 (60)
1	12 (34.3)
2	2 (5.7)
Gender (%)	
Male	27 (77.1)
Female	8 (22.9)
Primacy cancer (%)	
Prostate	25 (71.4)
Cervix	8 (22.9)
Rectum	2 (5.7)
Co-morbidities	
Liver cirrhosis	4 (11.4)
Coronary artery disease	2 (5.7)
Diabetes mellitus	8 (22.9)
Hypertension	10 (28.6)
Chronic kidney disease	4 (11.4)
End stage renal disease	1 (2.9)
Anti-coagulant use (%)	6 (17.1%)
Radiation dosage (%)	
<5000 cGy	3 (8.6)
>5000 cGy	32 (91.4)
Onset interval of CRP after radiotherapy, mean (±SD), months	12.3 (±6.5)
Prior medical therapy (%)	
Hydrocortisone enema	30 (85.7)
Formalin irrigation	2 (5.7)
Hyperbaric oxygen therapy	2 (5.7)
Argon plasma coagulation	1 (2.8)
Blood transfusion before RFA (%)	15 (42.9)
Symptoms (%)	
Rectal bleeding	35 (100)
Tenesmus	10 (28.6)
Diarrhea	4 (11.4)
Follow up time (±SD), months	18.6 (±12.4)

SD, standard deviation; ECOG, Eastern Cooperative Oncology Group.

**Table 2:** Statistics and analysis.

Variable	preRFA	postRFA	*p*
Hb level (3 months after RFA), *n* = 32	10.4 ± 2.0	11.0 ± 2.0	0.121
Hb level (6 months after RFA), *n* = 28	11.0 ± 2.3	11.8 ± 1.9	0.048
RTD score, *n* = 25	2.96 ± 0.2	0.85 ± 0.7	<0.0001

Hb, hemoglobin; RTD, rectal telangiectasia density; RFA, radiofrequency ablation.

**Table 3:** Adverse events after radiofrequency ablation (RFA) treatment.

Variable	Results
Hematochezia (%), interval (weeks)	25 (71.4%%), 7.4 ± 4.4
Anal pain (%)	12 (34.2%)
Anal stenosis (%)	2 (5.7%)
ER visit related to rectal bleeding	2 (5.7%)
Blood transfusion after RFA	1 (2.9%)

ER, Emergency room; RFA, radiofrequency ablation.

**Table 4:** Summary of literature regarding the use of radiofrequency ablation for chronic radiation proctitis.

Author, Country	Year	Patient Number	Primary Cancer (*n*)	Mean Age	RFA Settings (J/cm^2^)	Pre-RFA Hb (g/dL)	Post RFA Hb (g/dL)	Serious Adverse Events (*n*)	Success Rate (%) (Technical/Clinical)
Zhou, USA [14]	2009	3	Prostate (3)	72	10	-	-	0	100/100
Patel, USA [29]	2014	3	Prostate (3)	81.7	12	6.7	10	0	100/100
Pigo, Italy [30]	2014	4	Prostate (4)	71.3	12	-	-	0	100/100
Dray, France, Italy, Spain [31]	2014	17	Prostate (11) Cervix (3) Bladder (1) Anal (1) Uterine (1)	74	12 (*n* = 10); 15 (*n* = 7)	8.3	11.3	0	100/88.2
Rustagi, USA [12]	2015	39	Prostate (38) Urethral (1)	72.9	12	11.8	13.5	1	100/95.8
Markos, Croatia [32]	2017	5	Prostate (3) Uterine (2)	73	12	8.6	10.3	0	80/80
Chou, Taiwan [33]	2018	3	Prostate (2) Uterine (1)	68.7	12	-	-	0	100/100
Tang, Taiwan (This study)	2022	35	Prostate (25) Cervix (8) Rectum (2)	70.5	15	11	11.8	1	100/100

## Data Availability

Not applicable.

## Abbreviations

The following abbreviations are used in this manuscript:

APC	argon plasma coagulation
COPD	chronic obstructive pulmonary disease
CRP	chronic radiation proctitis
DM	diabetes mellitus
ESRD	end-stage renal disease
Hb	hemoglobin
HBO	hyperbaric oxygen
HTN	hypertension
LDLT	living donor liver transplantation
RFA	radiofrequency ablation
RTD	rectal telangiectasia density

## Appendix A

**Table A1:** Patient Characteristics.

No	Primary Cancer	Age/Sex	Co-Morbidity	Radiation Dosage (cGy)/Fractions	Onset Interval (Weeks)	Pre-RFA Hb (g/dL)	Post-RFA 3 Months Hb (g/dL)	Post-RFA 6 Months Hb (g/dL)	Pre-RFA RTD Score	Post-RFA RTD Score	Follow-Up Interval (Months)	Post-RFA Hematochezia Interval (Weeks)
1	Rectum	60/M	COPD, Liver cirrhosis	6000/30	12	15.1		14.2	3		6	6
2	Cervix	84/F	ESRD, DM, HTN, CAD	4500/25	11	9.4	11.6		3		3	Nil
3	Rectum	58/M	CAD	5800/29	15	13.4	12.7	12.9	3	2	38	8
4	Prostate	84/M		6660/37	12	8.8	4.5		3	1	3	8
5	Prostate	82/M	COPD	7600/38	12	13.6	12.8	13.3	3	1	30	8
6	Prostate	76/M		7600/38	16	10.4	11	11.4	3	1	7	2
7	Prostate	86/M	HTN	7000/35	12	13.8	13.1	14.4	3	0	52	24
8	Prostate	74/M	CKD	7600/38	4	8.1	13.2	13.8	3	1	42	12
9	Prostate	69/M		3625/5	9	14.3		14.4	3	0	10	2
10	Cervix	61/F		5000/25	10	10.6	10.4	10.4	3	1	9	4
11	Cervix	77/F		5000/25		11.6	11.2	10.9	3	0	18	8
12	Cervix	47/F		5000/25	10	8.8	9.3	10.3	3	2	8	6
13	Cervix	38/F		Lack	11	8.9	11.4		3	2	7	Nil
14	Prostate	68/M	HTN	7600/38	8	12.5	12.2	12.2	3	0	40	3
15	Prostate	71/M	Liver cirrhosis, HCC, DM, HTN	7600/38	4	10.3	11.7	11.5	3	1	22	Nil
16	Prostate	79/M	DM	7600/38	10	8.8	9.5	10.3	3	1	28	8
17	Prostate	81/M	Liver cirrhosis, HCC, DM, HTN	7600/38	10	10.5	9.8	10.6	3	0	29	6
18	Prostate	53/M	HTN	7600/38	6	13.2	12.7	12.9	3	0	25	8
19	Prostate	73/M		7600/38	15	7	7.7	8.4	3	2	26	12
20	Prostate	70/M	DM	7600/38	9	9.3	13.8	14.8	3	1	12	Nil
21	Prostate	66/M		7600/38	22	13.5	14.2	14.7	3	0	12	8
22	Prostate	80/M	DM	7200/26	12	8.8	11.8	12.1	3		15	4
23	Cervix	45/F		5000/25	10	10.8	12.5	12.5	3	0	36	4
24	Cervix	50/F		5040/28	16	12.1	11.8		3	0	23	Nil
25	Prostate	72/M	HCC	6400/32	7	9.5	9.1	6.5	3		2	3
26	Prostate	69/M		7600/38	9	11.7	10	8.6	2	1	24	Nil
27	Prostate	87/M		7600/38	13	12.1		10.7	3		8	Nil
28	Prostate	66/M	Liver cirrhosis s/p LDLT	3625/5	40	11.9	10.8	10.7	3		16	Nil
29	Prostate	80/M		7600/38		9.8	9.6		3		5	Nil
30	Prostate	79/M		Lack	12	6.7	8.9	10	3	2	19	10
31	Prostate	86/M		4500/25; 7560/17	12	14	12.4	13.4	3	1	12	8
32	Prostate	71/M	DM, CKD	7000/35	24	10.3	10.5	9.2	3	1	16	8
33	Prostate	71/M	CKD	7600/38	12	8.0	9.4		3		12	8
34	Cervix	78/F		Lack	9	9.0	10	10	3		18	8
35	Prostate	76/M	Liver cirrhosis, HTN, CKD	7000/35	12	8.9	11	12.9	3	1	18	Nil
					12.3 ± 6.5	10.7 ± 2.2	10.9 ± 1.9	11.7 ± 2.1			18.6 ± 12.4	

COPD, chronic obstructive pulmonary disease; ESRD, end-stage renal disease; DM, diabetes mellitus; HTN, hypertension; LDLT, living donor liver transplantation; Hb, hemoglobin; RTD, rectal telangiectasia density; RFA, radiofrequency ablation.
